# Citicoline in hypoxic ischemic encephalopathy in neonates: a randomized controlled trial

**DOI:** 10.1186/s13052-023-01452-5

**Published:** 2023-05-12

**Authors:** Abeer Salamah, Doaa El Amrousy, Mai Elsheikh, Mostafa Mehrez

**Affiliations:** 1 Pediatric Department, Kafr Elsheikh University, Kafr Elsheikh, Egypt; 2 Pediatric Department, Faculty of Medicine, Tanta University, Tanta, Egypt

**Keywords:** Hypoxic ischemic encephalopathy, Neonates, Citicoline, Seizures, Neurodevelopmental outcomes

## Abstract

**Background:**

Hypoxic-ischemic encephalopathy (HIE) is one of the major complications that can lead to death or disability in neonates. We assessed the effect of citicoline as a neuroprotector in neonates with moderate and severe HIE.

**Methods:**

This clinical trial was carried on 80 neonates with moderate to severe HIE who were not candidates for therapeutic cooling. They were subdivided randomly into two groups; citicoline treatment group which included 40 neonates who received citicoline 10 mg / kg /12 h IV for 4 weeks plus other supportive measures and the control group which included 40 neonates who were managed with placebo and the same supportive measures. All patients were evaluated for duration of mechanical ventilation (MV), need for inotropes, seizures (type, frequency, and duration), and duration of NICU. Cranial ultrasounds and brain magnetic resonance image (MRI) were performed for all included neonates after 4 weeks of treatment. Follow- ups of all neonates for the neurodevelopmental outcomes were done at 3, 6, 9, and 12 months.

**Results:**

There was a significant reduction in the number of neonates having seizures after discharge in the citicoline-treated group (2 neonates) compared to the control group (11 neonates). Cranial ultrasound and MRI findings at 4 weeks were significantly better in the treatment group compared to the control group. Moreover, neurodevelopmental outcome showed significant improvement at 9 and 12 months in the citicoline treated neonates compared to the control group. There was statistically significant reduction in the duration of seizures, NICU stay, inotrope use, and MV in the treatment group compared to the control group. Citicoline was well tolerated with no remarkable side effects.

**Conclusion:**

Citicoline could be a promising neuroprotector drug in neonates with HIE.

**Trial registration:**

The study was registered at ClinicalTrials.gov (NCT03949049). Registered at 14 May 2019, https://clinicaltrials.gov/ct2/show/NCT03949049

## Background

Hypoxic-ischemic encephalopathy (HIE) is a type of birth injury that leads to neonatal brain damage [1]. The incidence of HIE is 1.5 per 1000 live births in developed countries and varies between 2.3–26.5 per 1000 live births in developing countries [2].

Infants who experience moderate HIE have a 10% risk of fatality, and those who live have a 30% risk of disabilities. Infants with severe HIE have a 60% risk of fatality, and nearly all of the survivors experience disabilities [3].

Ischemia in the brain triggers a series of metabolic reactions that result in an influx of sodium (Na +) and calcium (Ca2 +) into cells resulting in the release of neurotransmitters like glutamate, causing cell excitotoxicity and the formation of free radicals that stimulate synthesis of nitric oxide (NO) which can react with superoxide causing subsequent brain injury [4, 5].

There are several treatment options used now for HIE as xenon, erythropoietin, melatonin, and stem cell therapy. The clinical use of these neuroprotective drugs in HIE is suboptimal due to inefficiency and /or treatment-induced undesirable side effects [6] (Table 1).

**Table 1:** Potential therapies of hypoxic ischemic encephalopathy and their mechanism of actions

Erythropoietin, darbepoietin	Favors neurogenesis; works as an antioxidant, anti-inflammatory, decreases apoptosis and excitotoxic cell injury
Stem cells	Stem cells modulate the immune system and affect long-term outcomes
Melatonin	Works as neuroprotective
Xenon	Antagonizes the N-methyl-D-aspartate mediated excitotoxicity decreasing calcium entry into the cell
Allopurinol	Works by inhibiting the enzyme xanthine oxidase, decreases reactive oxygen species, mitochondrial lysis, and cell death
Topiramate	Blocks the voltage-dependent sodium and calcium channels and also inhibits the excitatory glutamate pathway while enhancing the inhibitory effects of gamma-aminobutyric acid
Magnesium sulfate	Antagonizes the N-methyl-D-aspartate mediated excitotoxicity and decreasing calcium entry into the cell
Monosialogangliosides	protect against apoptotic injury
Endocannabinoids	Preclinical studies
Argon	Preclinical studies
Azithromycin	Preclinical studies

However, the only available effective neuroprotector so far is therapeutic hypothermia which can’t be used in several situations such as gestational age less than 36 weeks, birth weight less than 2000 g, and more than 6 h of age at the time of initiating therapeutic hypothermia [7]. Hence, finding an alternative safe, effective, and available method of neuroprotective therapy, especially in developing countries, is crucial.

Citicoline, refers to the exogenously supplied form of cytidine 5-diphosphocholine (CDP-choline), is a product of the rate-limiting step in the synthesis of phosphatidylcholine from choline. Citicoline is rapidly absorbed when taken orally because it is digested in the gut into cytidine and choline [8].

Citicoline could be a promising new neuroprotector in HIE as its mechanism of action helps in the regeneration of neuronal cells by several actions. As HIE induces cell apoptosis, citicoline provides neurorepair by inhibiting different steps of the ischemic cascade protecting the injured tissue against early and delayed mechanisms responsible for ischemic brain injury such as inhibition of glutamate accumulation, regeneration of the injured cell membrane, increasing brain plasticity and repair, increasing useful neurotransmitters such as dopamine and acetylcholine, and preventing free radicle formation [9, 10].

In this study, we assessed the effect of citicoline as a neuroprotector in neonates with moderate and severe HIE.

## Methods

This randomized clinical trial was carried out at neonatal intensive care units (NICU), pediatric departments of Tanta University and Kafrelsheikh University Hospitals during the period from May 2019 to December 2021 on eighty neonates suffering from moderate to severe HIE. Institutional ethical committee approval was obtained and written consent was taken from the guardians of all included neonates. The study was registered at ClinicalTrials.gov (NCT03949049).

Inclusion criteria: neonates with moderate to severe HIE according to Sarnat classifications [11] who did not meet therapeutic cooling criteria (postnatal age of more than 6 h, birth weight less than 2000 g, and gestational age less than 36 w).

Neonates with HIE who underwent therapeutic cooling, gestational age less than 34 weeks, major congenital malformations, or the presence of a known lethal chromosomal anomaly were excluded.

All children meeting the inclusion criteria were randomly assigned to one of the two groups:

- The citicoline treatment group: included 40 neonates with HIE who received citicoline 10 mg /kg /12 h IV within 6 h of admission for 4 weeks plus other supportive measures (including mechanical ventilation, inotropic support, and phenoparbitone for management of seizures if needed) in the NICU [12].
- The control group: included 40 neonates with HIE who were managed with placebo and the same supportive measures as indicated.

The random number list was generated by computer software. Allocation concealment was performed by sequentially numbered sealed opaque envelopes. Once the written consent was signed, the sealed opaque envelope was opened and the neonate was enrolled into the respective group by the doctor on duty who was not a part of the study. The outcome assessors and investigators were blinded to the treatment groups.

All patients enrolled in this study were evaluated for postnatal age, gestational age, Sarnat staging, capillary blood gases, duration of resuscitation, and urinary lactate /creatine ratio [13]. The duration of mechanical ventilation (MV) if needed, need for inotropes, seizures (type, frequency, and duration), and duration of NICU stay were recorded.

Conventional electroencephalogram (EEG) was performed for all included neonates by Nicolet EEG V32 and recording was done using caps including electrodes. Cranial ultrasounds and brain magnetic resonance images (MRI) were performed for all included neonates after 4 weeks of treatment. Images were blindly reviewed by a specialized pediatric neuroradiologist.

All the included neonates were followed up for 1 year to assess the developmental progress and the occurrence of seizures. Assessment of the development was performed at 3 months, 6 m, 9 m, and at 1 year using Griffiths mental development scales (GMDS) by a pediatric neurologist. Vital signs were monitored during the period of citicoline treatment and any side effects were also recorded. Griffiths mental development scales (GMDS): are used for the neurodevelopmental assessment of the included infants. Scores are estimated for each subscale, and an overall general quotient (GQ) score is then calculated. A GQ of ≥ 90 was considered normal, 80–89 was mild disability, 70–79 was moderate disability, and < 70 was severe disability [14].

The primary outcome was to evaluate the improvement of GMDS after citicoline treatment. The secondary outcomes were to evaluate the effect of citicoline on duration of NICU stay, duration of MV, duration of inotropes, seizure frequency and duration, and to assess side effects of citicoline.

### Statistical analysis of the data

The data was fed to the computer and analyzed using IBM SPSS software package version 20.0. (Armonk, NY: IBM Corp). The Kolmogorov- Smirnov was used to verify the normality of the distribution of variables. Continuous data were presented in the form of mean and standard deviation (SD) if normally distributed and in the form of median and range if skewed. Categorical data were presented in the form of numbers and percentages. Comparisons between groups for categorical variables were assessed using the Chi-square test. Student t-test was used to compare two groups for normally distributed quantitative variables. The Mann–Whitney test was used to compare two groups for non-normally distributed quantitative variables. The significance of the obtained results was judged at the 5% level.

## Results

**Fig. 1:**
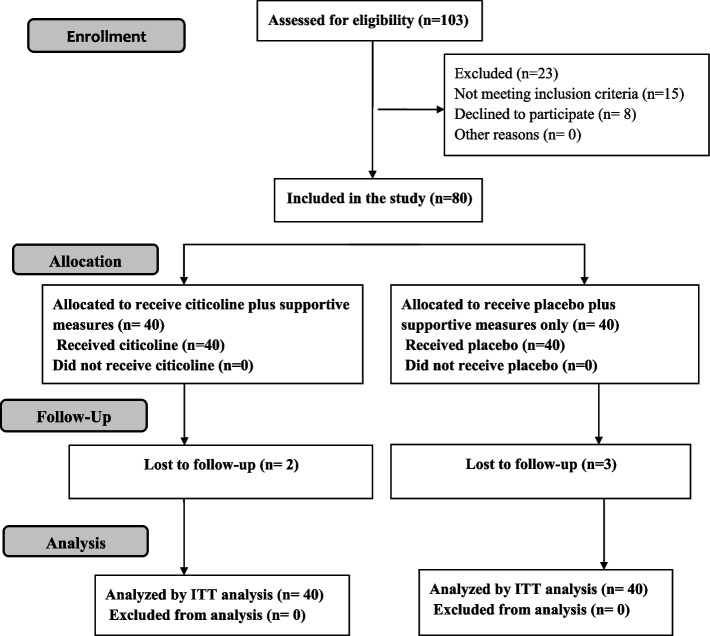
Flow diagram of the study

The flow diagram of the study was presented in Fig. 1. Eighty eligible neonates were enrolled in our study who was further subdivided into two groups; the treatment group (*n* = 40) and the control group (*n* = 40). We lost two patients in the citicoline group; one of them died at the age of 6 months with a severe chest infection and we lost contact with the other one, while we lost three patients in the control group who died at the ages of 3, 7, and 9 months. The treatment group had a mean GA of 38.3 ± 1.8 weeks, a mean birth weight of 2990 ± 670 g, and they were 20 males and 20 females, while the control group had a mean GA of 38.1 ± 1.7 weeks, a mean birth weight of 2850 ± 590 g, and they were 27 males and 13 females. The treatment group included 19 neonates with moderate HIE and 21 neonates with severe HIE, while the control group included 23 neonates with moderate HIE and 17 neonates with severe HIE. There were no statistically significant differences between the two groups according to gestational age, weight, sex, duration of resuscitation, pH, base deficit, Apgar score at 10 min, Sarnat staging, or urinary lactate / creatine ratio on admission. Moreover, EEG was comparable between the two groups (Table 2).

**Table 2:** Demographic, clinical, and laboratory data of the studied groups at admission before treatment

Parameters	Citicoline group	Control group	P
GA (weeks)	38.3 ± 1.8	38.1 ± 1.7	0.658
Birth Weight (gm)	2990 ± 670	2850 ± 590	0.331
Sex (male:female)	20:20	27:13	0.112
Duration of resuscitation (min)	11 (6–20)	9 (5–19)	0.068
Apgare score at 10 min	3.58 ± 1.26	3.60 ± 1.32	0.889
PH (first hour)	6.98 ± 0.11	7.01 ± 0.10	0.305
HCO3	12 (2–19)	12.5 (2–20)	0.012
Base deficit	16.13 ± 1.04	16 ± 0.93	0.679
Urinary Lactate/creatine ratio	0.79 ± 0.09	0.80 ± 0.09	0.468
Sarnat staging: N (%)			
-stage 2	19 (47.5%)	23 (57.5%)	0.370
-stage 3	21 (52.5%)	17(42.5%)	
EEG: N (%)			
-Normal continuous	22 (55%)	28 (70%)	0.554
-Low voltage	5 (12.5%)	2 (5%)	
-Burst suppression	5 (12.5%)	4 (10%)	
-Seizure activity	7 (17.5%)	4 (10%)	
-Paroxysmal generalized epileptiform	1 (2.5%)	2 (5%)	

*GA* gestational age, *HCO*_*3*_ bicarbonate, *EEG* electroencephalogram

The GQ of GMDS was significantly higher in the citicoline treated group compared to the control group starting from the age of 9 months. However, the GQ of GMDS was comparable in both groups at the ages of 3 and 6 months (Table 3).

**Table 3:** Comparison of Griffith’s mental developmental scales at 3, 6, 9, and 12 months

Parameters	Citicoline group	Control group	*P*
GQ at 3 months	82 ± 9	79 ± 10	0.342
GQ at 6 months	88 ± 7	80 ± 11	0.09
GQ at 9 months	91 ± 10	82 ± 11	0.01
GQ at 12 months	98 ± 13	83 ± 12	<0.001

*GQ* general quotient

There was a significant reduction of post-discharge seizures in the citicoline-treated group compared to the control group, as seizures persisted after discharge in only 2 neonates (5%) in the treatment group, while post-discharge seizures occurred in 11 neonates (27.5%) in the control group. Cranial ultrasound and MRI findings at 4 weeks were significantly better in the treatment group compared to the control group. Results showed that there was a statistically significant reduction in the duration of seizures, NICU stay, inotrope use, MV, and number of patients who needed inotropes and MV in the treatment group compared to the control group (Table 4).

**Table 4:** Different outcome in the studied groups

Parameters	Citicoline group	Control group	P
Number of patients with seizures during trteatment: N (%)	8 (20%)	15 (37.5%)	0.04
**Type of seizures: N (%)**			
-No seizure	32 (80%)	25 (62.5%)	0.092
-Focal	2 (5%)	4 (10%)	
-GTCs	6 (15%)	7 (17.5%)	
-Focal clonic	0 (0%)	1 (2.5%)	
-Myoclonic	0 (0%)	1 (2.5%)	
- Clonic	0 (0%)	1 (2.5%)	
- Focal & GTCs	0 (0%)	1 (2.5%)	
Seizure duration (min)	2 (0.2 – 15.0)	6 (1 – 15.0)	0.02
Number of patients with post discharge seizure	2 (5.0%)	11 (27.5%)	0.006
Duration of NICU stay (days)	25.58 ± 9.36	34.43 ± 13.53	0.001
Number of patients who needed inotropes	9 (22.5%)	20 (50%)	0.011
Duration of inotropes(hours)	12.0 (1.0 – 72.0)	24.0 (3.0 – 96.0)	0.032
Duration of MV (days)	10.85 ± 3.21	21.50 ± 6.64	< 0.001
**Cranial US on discharge: N (%)**			
-Normal - Abnormal	32 (80%) 4 periventricular leukoencephalopathy (10%) 4 periventricular leukomalacia (10%)	22 (55%) 9 periventricular leukoencephalopathy (22.5%) 8 periventricular leukomalacia (20%) 1 cortical atrophy (2.5%)	0.006
**MRI brain on discharge: N (%)**			
-Normal - Abnormal	30 (75%) 4 periventricular leukoencephalopathy (10%) 4 periventricular leukomalacia (10%) 2 cortical atrophy (5%) (1 of them had caudate nucleus changes)	19 (47.5%) 21 (52.5%) 9 periventricular leukoencephalopathy (22.5%) 8 periventricular leukomalacia (20%) 4 cortical atrophy (10%) (1 of them had focal changes in the lateral thalamus and lentiform nucleus)	0.001

*GTCs* generalized tonic clonic convulsions, *NICU* neonatal intensive care unit, *MV* mechanical ventilation, *US* ultrasound, *MRI* magnetic resonance imaging

Citicoline was well tolerated with no remarkable side effects on heart rate, respiratory rate, or blood pressure. Only four neonates experienced mild diarrhea with its use that stopped alone and didn’t necessitate treatment.

## Discussion

HIE is still one of the major causes of mortality and neurologic disability in neonates [2]. Despite well experienced knowledge and application of hypothermia in HIE in the developed countries, the availability of rapid intervention with cooling in cases of hypoxia within the first six hours of birth is not applicable in many patients due to delayed transfer. Furthermore, the availability of cooling is limited to tertiary centers only [15, 16]. Hence, the search for alternative effective neuroprotector drugs is crucial.

In this study, citicoline was tried as a neuroprotector drug in neonates with moderate to severe HIE in whom therapeutic cooling was not applicable due to several reasons and citicoline was found to have good neuroprotecting results in the treatment group compared to the control group. To our knowledge, this is the first clinical trial to use citicoline as a neuroprotector in neonates with HIE.

In this trial, we reported a significant reduction in post-discharge seizures in citicoline-treated group compared to the control group. Moreover, the duration of seizures was significantly shorter in citicoline treated neonates compared to the control group. Similar results were documented in a small pilot study performed by Khushdil et al. [17]. However, their results were limited by the small sample size and lack of a control group.

One of the important effects of citicoline intake is the decreased glutamate release after accumulation after brain hypoxia, with a subsequent reduction of seizure activity of the brain that is aided by a decrease in the number of glutamatergic synapses and extracellular glutamate concentration [18]. This was evidenced by the studies done on experimental animals using intraperitoneal injection of citicoline leading to increased seizure threshold for both tonic and clonic seizures [19]. Moreover, a similar study showed that using citicoline with an antiepliptic drug (valropate) reduced the dose needed to control seizures in comparison to the use of the antiepileptic agent alone [20].

Cranial ultrasound scanning and MRI brain in our study showed better results in the citicoline treated group compared to the control group after 4 weeks of treatment. Moreover, there was a significant improvement of neurodevelopmental outcomes at 9 and 12 m of age in the treatment group compared to the control group.

Several mechanisms can be responsible for the neuroprotective effects of citicoline in neonates with HIE. Citicoline is vital in the production of phospholipids which are necessary for the formation and regeneration of neuronal cytoplasmic and mitochondrial membranes following damage [20, 21]. Moreover, citicoline promotes recovery by enhancing synaptic outgrowth, endogenous brain plasticity and repair which helps to lessen acute brain damage and increase functional recovery in animal models of stroke, even when given many hours after the ischemic event which was one of the determinants of its trial in neonates with hypoxic insult after 6 h of birth [22].

Part of the citicoline protective and restorative effects on the central nervous system (CNS) is by increasing Na + /K + ATPase activity, decreasing lipid peroxidation, improving neuroplasticity, and increasing the synthesis of neurotransmitters such as acetylcholine (Ach), and dopamine [23, 24].

Moreover, one of the explanations of functional improvement after citicoline use in post stroke patients is the ability of citicoline to do mobilization of endothelial progenitor cells from the bone marrow of stroke patients, these cells secrete growth factors. These growth factors when tried experimentally as treatment have been shown to improve cognitive recovery in conditions such as traumatic brain injury and experimental stroke [25, 26].

Another randomized, placebo-controlled, double-blind clinical study had also showed benefits with short-term use of citicoline for 6 weeks in acute ischemic stroke with significant recovery at 12 weeks when compared to placebo with no differences with placebo in adverse events [27].

This study showed that there was a statistically significant reduction in the duration of NICU stay, inotrope use, and MV in the treatment group compared to the control group. Due to its broad spectrum of activity on neural tissue, citicoline is considered a promising neuroprotector drug. Furthermore, citicoline is reported to be a safe drug with no serious adverse effects [28]. Due to lack of clinical research on citicoline use in neonates with HIE, the results of this study can be considered an important step in the direction of the use of a promising new neuroprotective drug in neonatal HIE, especially in underdeveloped countries.

The main limitations of this study are being a two-center study with a limited number of patients and short follow-up period. Thus, larger multicenter studies with higher number of patients including the trial of different doses of citicoline and longer follow up are needed to prove the potential efficacy of citicoline in neonatal HIE. Moreover, we preferred 4 weeks for scanning by MRI rather than 2 weeks secondary to some technical difficulties like transportation of mechanically ventilated children as some patients enrolled in the study were mechanically ventilated and others were too unwell and unstable to be scanned within the first weeks after birth.

## Conclusion

Citicoline could be a promising neuroprotective drug in neonates with HIE.

## Data Availability

Data are available from the corresponding author on reasonable requested.

## Abbreviations

CDP-choline: Cytidine 5-diphosphocholine
EEG: Electroencephalogram
GQ: General quotient
GMDS: Griffiths mental development scales
HIE: Hypoxic ischemic encephalopathy
MRI: Magnetic resonance imaging
NICU: Neonatal intensive care unit
NO: Nitric oxide
MV: Mechanical ventilation
